# The Order of Mercy

**Published:** 1913-01-11

**Authors:** 


### The Order of Mercy.

The King, Sovereign of the Order of Mercy, has sanctioned the award of the Order to the following:—

Lady Presidents.—Lady Idina Hythe, East Sussex; Miss Campbell of Auchmannoch, Hythe.

Vice-Presidents.—Mr. W. R. Graham, S.E. Essex; Mr. P. Hollingworth, Southwark-Bermondsey; Lieutenant-Colonel King, N. St. Pancras; and Mr. Arthur Tite, Ealing.

Lady Vice-Presidents.—The Countess of Bradford, St. George's, Hanover Square; the Countess of Malmesbury, Hampshire; Mrs. S. N. Brain, N. St. Pancras; Mrs. Henry Burton, Enfield; Mrs. Phipps Carey, Westminster; Mrs. M. Hildred L. Causton, Battersea; Mrs. Crawshay, Oxfordshire; Miss M. Draper, Fulham; Mrs. Girvin, Lewisham; Mrs. E. Greenwell, E. Marylebone; Mrs. Hillard, Hammersmith; Mrs. E. G. Hime, S. Kensington; Mrs. Inglis, Fulham; Mrs. Pizey, Epping; Miss Pryce, Luton; Mrs. Cortlandt Thompson, S. Paddington; Mrs. Tuohy, Ealing; and Mrs. Willis, S. Kensington.

Members.—Mr. W. Bertram, City of London; Mr. T. N. Cox, Deptford-Brockley; Mrs. Alen, Harwich; Mrs. Bates, Chelsea; Miss E. Blackwell, Brentford; Mrs. G. Chandler, Tonbridge; Mrs. Chope, Chelsea; Mrs. Davidson, Bethnal Green; Miss Edwards, Sevenoaks; Miss Faudel-Phillips, St. George's, Hanover Square; Miss M. Hart, S. Kensington; Miss James, Dulwich-Norwood; Mrs. Lohden, Dulwich-Norwood; Mrs. Marson, Bucks; Miss J. Montagu, Fulham; Miss Moore, Epsom-Esher; Miss Marion Edith Narborough, Woolwich; Mrs. Parsons, Dulwich-Norwood; Mrs. Pearson, W. Islington; Mrs. H. Samuel, S. Paddington; Miss Ada Sorsbie, Colchester; Miss A. Tooth, W. Marylebone; Mrs. Tufton, N. Paddington; and Mrs. Van der Bergh, S. Kensington.

